# EPIDERMOLYSIS BULLOSA: A SERIES OF 12 PATIENTS IN KASHMIR VALLEY

**DOI:** 10.4103/0019-5154.70668

**Published:** 2010

**Authors:** Seema Qayoom, Qazi Masood, Javeed Sultan, Iffat Hassan1, Majid Jehangir, Yasmeen J Bhat, Taseer Bhat, Muzamil Chisti

**Affiliations:** *From the Department of Dermatology, STD & Leprosy, SKIMS Medical College, Bemina, India*; 1 *From the Department of Government Medical College, Srinagar, India*

**Keywords:** *Blisters*, *epidermolysis bullosa*, *Nikolsky*, *sign*

## Abstract

**Background:**

Epidermolysis Bullosa (EB) is a genetically determined mechano-bullous disorder of the skin encompassing a group of conditions that share skin fragility as a common feature.

**Materials and Methods:**

Twele patients with Epidermolysis Bullosa from Kashmir valley are reported.

**Results:**

Our series included 12 patients, 5 males and 7 females. Features were consistent with EB simplex in 8 patients, EB pruriginosa in 2 patients, generalized atrophic benign EB in one patient and EB acquista in one patient.

**Conclusion:**

EB is a rare, genetically determined, blistering disorder affecting both males and females with predominant involvement of hands and feet. In the absence of specific therapy, treatment mainly involves avoidance of provoking factors, prevention and treatment of complications.

## Introduction

Epidermolysis Bullosa (EB) is a group of rare disorders, mainly inherited, characterized by blistering and erosions of the skin and mucous membranes resulting from slight mechanical trauma. The inherited disorders occur due to defects in the genes encoding various proteins like collagen and keratin, which are essential for mediating adherence of the epidermis to the underlying dermis.[1–3] In view of the rarity of this disorder, we report 12 cases of EB that presented to the department of dermatology of SMHS hospital between November 2004 and February 2006.

## Materials and Methods

Our series comprised of 12 patients of EB attending the department of Dermatology of SMHS Hospital, Srinagar, between November 2004 and February 2006. A detailed history was taken from each patient with special emphasis on photosensitivity, drugs, family history, consanguinity and symptoms suggestive of collagen vascular disease or any congenital infection. A thorough systemic and cutaneous examination was performed in each case. Routine investigations like complete blood counts, kidney function tests, liver function tests, blood sugar, urine examination along with collagen vascular profile, cryoglobulins, porphyrin profile and serum IgE levels were done in all patients. In each patient Tzanck smear was taken to exclude acantholysis. Diagnosis was confirmed by histopathological and direct immunofluoresence examination of the skin biopsy specimens. Electron microscopic examination could not be done due to the non-availability of this facility in our hospital.

### Cases 1, 2 and 3

**Figure 1:**
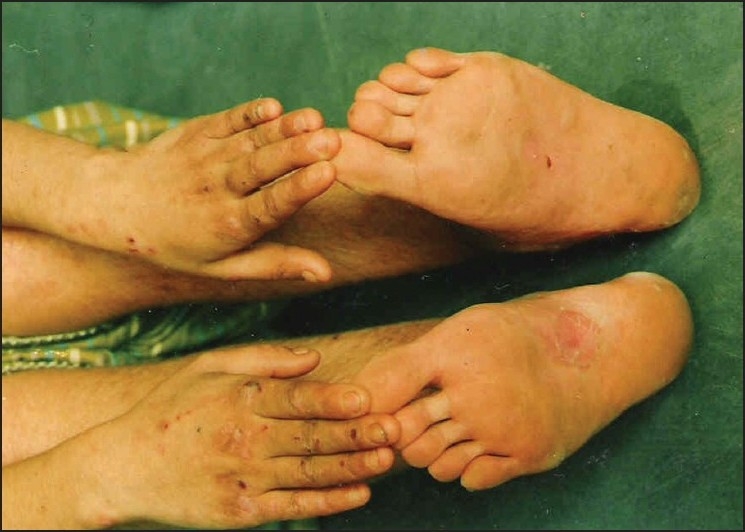
Blisters on hands and feet of patient 1

The first two cases were brothers aged 11 years and 15 years while the third was a 15 year old female. History of consanguinity was present in all. Spontaneous blistering at friction sites developed within first month of life. Subsequently, with crawling and walking, blisters started appearing on the hands and soles [Figure 1]. These blisters would rupture spontaneously leaving behind atrophic scars. Family history was non-contributory. Serum IgE levels were normal. Tzanck smear was negative. Histopathological examination of the skin biopsy specimen was consistent with EB simplex while direct immunoflourescence examination was negative in all three.

### Cases 4 and 5

**Figure 2:**
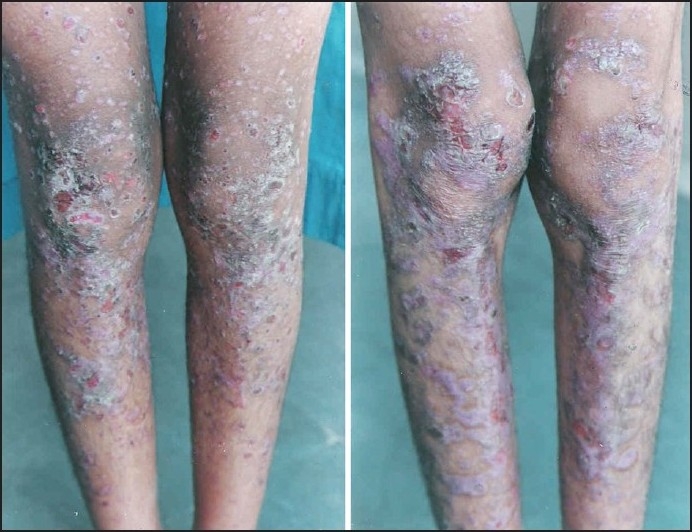
Blisters, scars and milia on the legs of the two cousins with EB pruriginosa

These were females 10 and 11 years of age, both born of consanguineous marriage. History of spontaneous blistering on pressure points since early childhood was present in both. Blisters would appear on toes, ankles, knees, elbows and eventually on abdomen and trunk. Crusting and atrophic scarring were present at the sites of blisters [Figure 2]. Partial loss of finger and toe nails was present in both. The mucosae were spared in both. Serum IgE levels were markedly raised, 1700 and 3000 IU respectively. Tzanck smear was negative. Histopathological examination of skin biopsy specimen was consistent with EB pruriginosa while direct immunoflourescence examination was negative in both. Both patients partially responded to oral dapsone, cyproheptadine and topical steroid-antibiotic combination.

### Cases 6, 7 and 8

Case 6 was a 35-year-old male, born of non-consanguineous marriage, who presented with a history of spontaneous blistering since early childhood. The patient had no other significant history. He was married to one of his first cousins and had two kids. Both their kids (males) had spontaneous blistering. All of the three had multiple flaccid blisters, mostly on fingers and toes. No atrophic scarring, nail changes or dental abnormality were seen in either of the three. Nikolsky’s sign was negative. Tzank smear was also negative. With histopathological examination of skin biopsy revealing intra-epidermal blistering and negative direct immunofluorescence study, a diagnosis of EB simplex was made.

### Case 9

**Figure 3:**
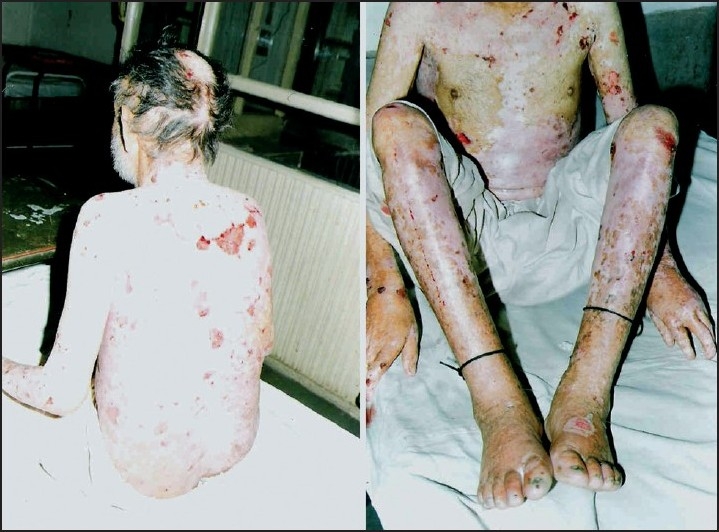
Blisters and scarring in patient with GABEB

The patient was a 66-year-old male non-smoker, normotensive, and non-diabetic. He presented with spontaneous blistering at frictional sites since early adolescence. There was also history of dysphagia. He was emaciated and had pallor. Cutaneous examination revealed multiple flaccid blisters filled with hemorrhagic fluid located on shins, elbows, trunk, groins and buttocks [Figure 3]. Atrophic scarring was also present. Mucosal erosions were present on hard palate along with enamel defects. There was scarring alopecia with sparse scalp hair and partial loss of toe nails. Patient had a hemoglobin of 6 gm/dl with peripheral blood film showing hypochromic microcytic cells. An upper gastrointestinal endoscopy was done, which revealed a stricture involving the upper third of the esophagus. A clinical impression of generalized atrophic benign EB (GABEB) with iron deficiency anemia was made.

### Case 10

**Figure 4:**
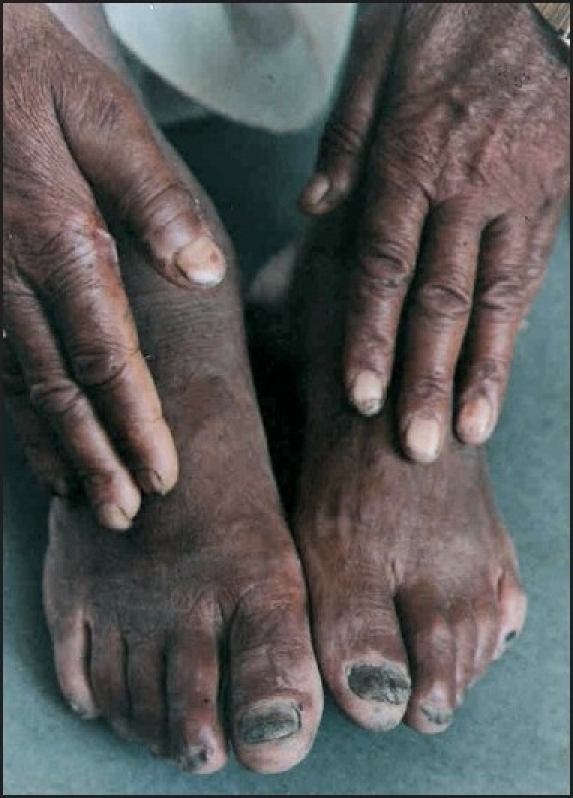
Blisters on hands and feet in EB acquista

This was the case of a 55-year-old male with end-stage renal disease on chronic ambulatory peritoneal dialysis. He had a history of spontaneous blistering on palms and soles of one month duration. There was no history of photosensitivity or any urticarial rash preceding the blistering. Examination revealed pallor with multiple fluid filled flaccid blisters on fingers and feet [Figure 4]. Nikolsky’s sign was negative. Investigations revealed a hemoglobin of 7.8 gm% with hypochromic microcytic cells in peripheral blood film. Blood urea was 101 mg% and creatinine was 10.9 mg%. Urine examination revealed albuminuria with granular casts. Liver function tests, blood sugar, collagen vascular profile and coagulogram were normal. Tzanck smear and direct immunofluoresence study were negative while histopathological examination revealed features consistent with EB acquista.

### Case 11

This was an eight-year-old boy, born of consanguineous marriage, with a history of spontaneous blistering on friction sites since early infancy. Mental and motor milestones of development were normal. There was no history of photosensitivity and family history was noncontributory. Cutaneous examination revealed normal mucosae, dentition, nails and scalp. Multiple atrophic scars were present on shins, trunk and abdomen. Tense fluid filled blisters were present on acral parts. Nikolsky’s sign was negative. Tzanck smear and direct immunofluoresence study were negative while histopathological examination revealed features consistent with EB simplex.

### Case 12

This was an 18-year-old female, born of consanguineous marriage, with a history of spontaneous blistering of frictional sites from infancy. There was no history of photosensitivity. There were two sibling deaths in the family, but both had apparently normal skin. Porphyrin profile and collagen vascular profile were normal. Nikolsky’s sign was negative. Tzanck smear and direct immunofluoresence study were negative while histopathological examination revealed features consistent with EB simplex.

## Observations and Results

Our case series included 12 patients, five males and seven females. Ten patients were between eight and 18 years of age while only two were above 50 years of age. In four patients there was history of blistering from the first three months of life. Positive family history was present in five patients while history of consanguinity was present in seven patients, including those four in whom blistering started from the first three months.

Clinico-pathological features were consistent with EB simplex in 8 patients, with EB pruriginosa in two patients and generalized atrophic benign EB in one patient while one patient with chronic renal failure was diagnosed as EB acquista. Hands and feet were involved in all the 12 patients, trunk in seven, nails in three and mucosae in only one patient. The serum IgE levels were markedly raised in the two patients with EB pruriginosa while renal function tests were deranged in the patient with EB acquista [Tables 1 and 2].

**Table 1:** Clinical characteristics of patients

Clinical type	Consanguinity	Age of onset (years)	No. of patients	Family history of similar disorder	Any other association	IgE levels	Deranged renal functions
EB simplex	+	3-30	4	+	–	Normal	−
		4-5	4				
EB pruriginosa	+	6-7	2	+	–	Markedly raised in both	−
Dystrophic EB (GABEB)	+	10	1	−	Dysphagia, Bleeding P/R	Normal	−
EB acquista	−	54	1	−	Chronic renal disease	Normal	+

**Table 2:** Distribution of lesions

Site of lesions	No. of patients
Cutaneous	
Hands	12
Feet	12
Face	None
Scalp	1
Trunk, arms	7
Mucosae	1
Nails	3

## Discussion

EB is a heterogeneous group of rare disorders characterized by blistering of the skin and mucosae upon exposure to mechanical stress. It was first described as ‘erblichen pemphigus’ by von Hebra in 1870.[4] The present name ‘epidermolysis bullosa hereditaria’ was coined by Koebner[5] in 1886; while characterization into major groups was first done by Pearson in 1962.[6]

Based on the level of dermoepidermal separation at the basement membrane (BM) zone, three major groups have been identified - simplex, junctional and dystrophic.[2,3,6,7] The level of separation in EB simplex is intraepidermal, in junctional EB it is at lamina densa and in dystrophic EB it is below the basement membrane.[1–3] Autosomal dominant as well as autosomal recessive forms of EB exist within each of the three major types. The mode of inheritance is mostly autosomal dominant in EB simplex and autosomal recessive in junctional EB.[2,3,7,8] The clinical severity of the different forms of EB varies widely and internal organs may also be involved.[2] The incidence and prevalence data from various countries suggests that there is no gender, racial, ethnic or geographical predilection for EB with EB simplex being the most frequent form of EB.[9]

In our patients, diagnosis was based on history, clinical examination and histopathology due to the non-availability of electron microscopy facility in our hospital. Hands and feet were involved in all the patients, consistent with the fact that sites subjected to frequent trauma and friction are predominantly involved in EB.

Trunk was involved in seven patients while nails were involved in three patients. Mucosal involvement was seen in only one patient. None of the patients had abnormal porphyrin profile or any serological marker suggestive of collagen vascular disorder. In both patients of EB pruriginosa, the serum IgE levels were markedly raised.

As there is no specific therapy at present for inherited EB, management mainly revolves around protection, avoidance of provoking factors, prevention and treatment of complications. In future, gene therapy may become possible for at least some types or subtypes of EB.[10,11]

As far as the management of our patients was concerned, patients with EB simplex were treated with topical antibiotics, vitamin E orally and advised about soft padded footwear. The patient of GABEB was screened for internal malignancy which was ruled out. He received topical steroids and antibiotics besides iron supplements. Patients of EB pruriginosa were started on dapsone, cyproheptadine orally, besides topical steroids. The response to treatment in them, however, remained unsatisfactory.
